# Proxy Responses for Mass Drug Administration Coverage Surveys: The Trends and Biases When Others are Allowed to Respond

**DOI:** 10.4269/ajtmh.21-0817

**Published:** 2021-10-25

**Authors:** Rini Jose, Roland Bougma, Franck Drabo, Edridah Muheki Tukahebwa, Square Mkwanda, Katherine Gass

**Affiliations:** ^1^ Department of Epidemiology and Biostatistics, Dornsife School of Public Health, Drexel University, Philadelphia, Pennsylvania;; ^2^ Direction de la Protection de la Santé de la Population, Ministère de la Santé, Ouagadougou, Burkina Faso;; ^3^ Vector Control Division, Ministry of Health, Kampala, Uganda;; ^4^ Lymphatic Filariasis Control Program, Ministry of Health and Population, Lilongwe, Malawi;; ^5^ Neglected Tropical Disease Support Center, Task Force for Global Health, Decatur, Georgia

## Abstract

Coverage surveys for mass drug administration (MDA) rely on respondent recall and often permit proxy responses, whereby another household member is allowed to respond on behalf of an absent individual. In this secondary analysis of coverage surveys in Malawi, Burkina Faso, and Uganda, we explore the characteristics of individuals who require proxy responses and quantify the association between proxy responses and reported drug coverage. The adjusted logistic regression model found that men 11–39 years and women 11–18 years who were eligible for MDA had greater odds of requiring a proxy response compared with ineligible men and women in the same age groups. A hierarchical multivariable analysis found that proxy responses had 1.70 times the odds of reporting ingestion of MDA drugs compared with first-person responses, controlling for age and sex (95% CI: 1.17, 2.46). This finding is surprising, given that individuals absent during a coverage survey may also have been absent during the MDA, and suggests that proxy responses may be leading to an inflation of survey estimates of drug coverage. This study highlights the possibility for recall bias in proxy responses to MDA coverage; however, excluding absent individuals from coverage surveys would introduce a new bias. Further research is necessary to determine the best method for obtaining information on drug coverage when individuals are absent.

## INTRODUCTION

Neglected Tropical Diseases (NTDs) are a group of parasitic, viral, and bacterial diseases prevalent among the world’s poorest populations in approximately 149 countries, affecting more than one billion people. These illnesses, when left untreated, can result in serious health and socioeconomic consequences, including impaired childhood growth and development, hindered economic prosperity, and the risk of life-long morbidity.^1^^–^^3^ Five of the 17 NTDs recognized by the WHO can be controlled through mass drug administration (MDA). MDA is the regular distribution of single-dose preventative chemotherapy drugs to an entire at-risk population.

If MDA is delivered repeatedly and with adequate coverage of the at-risk population, it can reduce the prevalence of infection and, in some cases, lead to elimination.^4^ Through MDA, the WHO hopes to effectively control and even eliminate these NTDs.^5^ The WHO and country programs use drug coverage as the core indicator to monitor the effectiveness of MDA and gauge progress on their control and elimination goals.^6^ Drug coverage is typically calculated by aggregating the number of people reported to have swallowed the drug(s) on drug distributors’ tally sheets or registers, divided by the at-risk population, which is often based on census estimates. This is referred to as “administrative coverage” and is the figure routinely reported for each implementation unit by national programs. While administrative coverage is quick to calculate and is computationally simple, this measure can be prone to certain biases and measurement errors.^7^ Incomplete reporting, data aggregation errors, or unreliable denominator estimates can bias the measure of administrative drug coverage, resulting in an inaccurate assessment of the success of an MDA.^8^ Population-based coverage surveys are independent random assessments of drug coverage that don’t suffer from these same biases.^9^ One major criticism of using population-based drug coverage surveys, however, is the issue of recall bias.

Recall bias refers to the ability to correctly recall whether an individual swallowed the medication, affecting one’s ability to correctly respond to survey questions related to coverage.^4^ This potential issue is further complicated when we consider proxy responses. Proxy responses are responses provided on behalf of someone who is absent, or unable to provide a reliable response, at the time the surveys are administered. Examples of proxy response include a mother answering the survey on behalf of her underage children or a woman answering on behalf of her spouse who is away from the home.^9^ Many survey practitioners accept proxy responses for ease of measurement and to avoid creating bias by limiting survey responses to those present at the time of the survey; however, proxy responses and the potential recall bias they induce have rarely been formally tested.^6^ This study takes advantage of data from a multi-country operational research study by Gass et al., aimed at comparing statistical methods for conducting population-based coverage surveys to conduct a secondary analysis assessing the characteristics of proxy responses compared with self-reported responses to questions of drug coverage.^10^

## MATERIALS AND METHODS

The data used in this study includes coverage surveys for lymphatic filariasis in Burkina Faso and Malawi, as well as a coverage survey following an integrated MDA for lymphatic filariasis, onchocerciasis, schistosomiasis, soil-transmitted helminthiasis, and trachoma in Uganda. The original purpose of the multi-country study was to compare three different sampling methods for coverage surveys by conducting three independent coverage surveys in each country. A full description of the study sites and coverage survey methodologies have been presented elsewhere.^10^ The present study is a secondary analysis of the original data, to determine what demographic characteristics affect the probability of a proxy response and to elucidate the relationship between proxy responses and drug coverage estimates.

### In-country team members.

In each country, survey data was collected by two surveyors: one record keeper, in charge of recording the time of arrival and the survey responses in a handheld electronic device; and one interviewer, in charge of interviewing the respondents and showing examples of medication offered during the MDA. At least one person in each pair was familiar with the study area and spoke the local language. Each country had a principal investigator, responsible for supervising the correct implementation of the various sampling methods, assessing the feasibility of each sampling method, and ensuring adherence to the study protocol.

### Study area and sampling design.

Districts in Burkina Faso, Malawi, and Uganda were primarily rural with little seasonal migration and were selected to be as similar as possible with regard to population density, area size, and endemicity of NTDs.^10^ The three separate survey methodologies used in each country included: the Expanded Program on Immunization’s (EPI) cluster survey methodology, probability proportionate to estimated size sampling using segmentation and enumeration to select households (PSS), and lot quality assurance sampling—this latter method was dropped from the current analysis due to a lack of sufficient household-level data. In both the EPI and PSS methodologies, 30 clusters were chosen from among all villages or census enumeration areas within the district using PSS. For the EPI approach, within each selected cluster, a direction was selected randomly, by standing at a central point in the cluster and spinning a bottle. Team members enumerated all households from the direction of the bottle spin to the periphery of the cluster, and randomly selected a number to determine which household would be the first selected for interview. The second household selected was the household whose front door was nearest that of the first household; the third household was the household whose front door was nearest that of the second household (excluding households already included in the study). This “nearest neighboring” household approach continued until the required sample size per cluster was met. For the PSS approach, each cluster was first divided into segments of approximately 50 households and a single segment was randomly selected. A fixed fraction of all households within the selected segment was included in the study. In both the EPI and PSS methods, all members living in the selected households, regardless of their availability at the time of the survey, were considered eligible for inclusion in the study.

In Malawi and Burkina Faso, each method was conducted independently in a separate district. Districts within the same country were chosen to have similar characteristics with regards to NTD endemicity, population density, and geography. In Uganda, all three survey methods were conducted as independent surveys in the *same* district, with the surveys conducted sequentially starting with PSS, followed by lot quality assurance sampling, and then the EPI method. In Malawi and Burkina Faso coverage of albendazole and ivermectin was assessed with a single question (e.g., “did you swallow the albendazole and ivermectin offered during the recent MDA?”). The Ugandan survey, in contrast, was an integrated assessment of multiple drugs (albendazole, ivermectin, praziquantel, and azithromycin). Each participant in the Uganda survey was asked whether they ingested each drug as a separate question.

### Eligibility and exclusion criteria.

All individuals who were living in the household during the time of the last MDA were eligible for the coverage surveys, regardless of whether they were eligible to receive the drugs at the time of the MDA. Individuals are deemed ineligible for lymphatic filariasis MDA with ivermectin if they are < 5 years old, pregnant women, in the first week of breastfeeding, and the severely ill. Participants were excluded from the present analysis if they responded “unsure” when asked if they swallowed the drug(s). Children 10 years and younger were also excluded from the analysis, as caretakers were required to provide a proxy response on their behalf and the present study is concerned with proxy responses provided on behalf of absent adults. To avoid assessing duplicate observations of individuals in Uganda, only ivermectin coverage was assessed; whereas ivermectin and albendazole were assessed jointly in Burkina Faso and Malawi.

### Ethics.

Ethical clearance from the local institutional review boards was sought in advance of each study. In Burkina Faso, ethical clearance was granted from the Ethical Committee for Health Research in the Ministry of Health. In both Malawi and Uganda, the Ministries of Health considered the coverage evaluation survey to be part of routine public health program practice and each sent a formal letter indicating that ethical approval was not necessary for the study. Permission to conduct the survey was obtained from community leaders on arrival in each primary sampling unit (PSU), and all participants gave verbal consent before participating in the survey.

### Variables of interest.

The main outcome variable was drug coverage status, a binary variable to denote whether the individual ingested the drug. The primary predictor variable was proxy response status, a binary variable denoted as “yes” when someone responded on behalf of the subject in question (i.e., a proxy response was necessary) or “no” for self-report. Throughout the remainder of this manuscript, the term “respondent” denotes the person who is providing the response, while “subject” refers to person for whom they are responding. When a person self-reports (i.e., proxy response = “no”) the respondent and subject are the same person.

Covariates considered in the analysis were: age of subject (recorded as a continuous variable, but later categorized as adolescents aged 11 to 18, young adults aged 19 to 39, and older adults 40+ years old), sex of subject (female or male), and eligibility for MDA (yes or no). District and country were also considered in the analysis.

### Data analysis.

All data cleaning and analyses were performed using SAS version 9.4 (Cary, NC). First, descriptive analyses (e.g., χ^2^ tests of association) were performed on all variables of interest, initially stratifying by drug coverage and then proxy response status. For cells with counts less than five, Fischer’s exact test was used. Finally, we fit two generalized linear mixed models. To account for the design of the study and the likely correlation between individuals living in the same cluster within the same districts, we included nested random intercepts for both district and cluster in both models. Initially, we assessed interaction, then fixed effects.^11^^,^^12^ The first model fit used proxy response status as the dependent variable and included demographic characteristics as response variables, as well as interaction terms of interest. The second and final model was fit to determine associations between drug coverage status and proxy response status, controlling for demographic characteristics and evaluating interaction terms of interest. Models in which the dependent variable was drug coverage were limited to eligible individuals, as ineligibility perfectly predicted a negative response to the question of drug coverage. Models in which the dependent variable was proxy response status included individuals who were both eligible and ineligible. We assessed interaction using likelihood ratio testing and backward elimination procedures. Random intercepts were evaluated using the COVTEST option to evaluate the independence and significance of random effects. For all models, we checked for collinearity by assessing the condition indexes and variance decomposition proportions. For all analyses, results were reported at the α = 0.05 level of significance.

## RESULTS

### Demographic characteristics.

A total of 6,261 subjects were interviewed to assess MDA coverage across the six surveys included in this analysis. Of these subjects, 5,932 were reportedly eligible for MDA, 132 were missing their eligibility status, and 197 were reported as ineligible for MDA. Among the subjects surveyed, 5,049 reported ingesting the drug(s) while 1,212 reported not ingesting the drug. The majority of responses (3,809; 60.8%) recorded were self-reported; 39.2% of responses came from a proxy respondent. Among subjects who self-reported, 76.5% reported taking the drugs, while among subjects with a proxy response 87.0% reported taking the drugs. Reported coverage was similar by sex. Young adults were least likely to report ingesting the drug (77.5%) compared with adolescents (82.4%) and older adults (83.6%) (Table 1).

**Table 1:** Demographic characteristics of subjects responding to a coverage survey for lymphatic filariasis, stratified by reported ingestion of the drug(s)

	All responses* (*N* = 6,261)		Did not ingest drug(s)† (*N* = 1,212)		Ingested drug(s)† (*N* = 5,049)		Chi-square test of association
	n	%	n	%	n	%	χ^2^ (*P* value)
Response status							105.39
							(*P* < 0.0001)
Proxy	2,452	39.2	318	13.0	2,134	87.0	–
Self-report	3,809	60.8	894	23.5	2,915	76.5	–
Eligible							1,446.58
							(*P* < 0.0001)
Yes	5,932	94.8	883	14.9	5,049	85.1	–
No	197	3.2	197	100.0	0	0.0	–
Missing	132	2.1	132	100.0	0	0.0	–
Age							29.93
							(*P* < 0.0001)
Adolescents, age 11 to 18	2,030	32.4	357	17.6	1,673	82.4	–
Young adults, age 19 to 39	2,628	42.0	592	22.5	2,036	77.5	–
Older adults, age 40+	1,603	25.6	263	16.4	1,340	83.6	
Sex							0.12
							(*P* = 0.7341)
Female	3,349	53.5	643	19.2	2,706	80.8	–
Male	2,912	46.5	569	19.5	2,343	80.5	–
Country							1,016.58
							(*P* < 0.0001)
Burkina Faso	2,297	36.7	111	4.8	2,186	95.2	–
Malawi	2,147	34.3	312	14.5	1,835	85.5	–
Uganda‡	1,817	29.0	789	43.4	1,028	56.6	–

* Column percentages reported, that is, percent of total responses.

† Row percentages reported, stratified by drug coverage.

‡ Individuals in Uganda were asked about albendazole, ivermectin, praziquantel, and zithromax separately. To avoid duplicate responses, only ivermectin responses were included.

Reported drug coverage varied considerably across countries, with 95.2% of subjects in Burkina Faso reported as ingesting the drugs, compared with 85.5% in Malawi and 56.6% in Uganda. After stratification, proxy response status, eligibility, age, and country were all strongly associated with drug coverage status (Table 1). Similarly, drug coverage, eligibility, age, sex, and country were all strongly associated with proxy response status (Table 2).

**Table 2:** Demographic characteristics of subjects responding to a coverage survey for lymphatic filariasis, stratified by proxy response status

	All responses* (*N* = 6,261)		Self-reported† (*N* = 3,809)		Proxy response† (*N* = 2,452)		Chi-square test of association
	n	%	n	%	n	%	χ^2^ (*P* value)
Drug coverage							105.39
							(*P* < 0.0001)
Yes, ingested	5,049	80.6	2,915	57.7	2,134	42.3	–
No, did not ingest	1,212	19.4	894	73.8	318	26.2	–
Eligible							186.99
							(*P* < 0.0001)
Yes	5,932	94.8	3,512	59.2	2,420	40.8	–
No	197	3.2	165	83.8	32	16.2	–
Missing	132	2.1	132	100.0	0	0.0	–
Age							104.60
							(*P* < 0.0001)
Adolescents, age 11 to 18	2,030	32.4	1,053	51.9	977	48.1	–
Young adults, age 19 to 39	2,628	42.0	1,684	64.1	944	35.9	–
Older adults, age 40+	1,603	25.6	1,072	66.9	531	33.1	–
Sex							149.53
							(*P* < 0.0001)
Female	3,349	53.5	2,273	67.9	1,076	32.1	–
Male	2,912	46.5	1,536	52.7	1,376	47.3	–
Country							244.59
							(*P* < 0.0001)
Burkina Faso	2,297	36.7	1,107	48.2	1,190	51.8	–
Malawi	2,147	34.3	1,480	68.9	667	31.1	–
Uganda‡	1,817	29.0	1,222	67.3	595	32.7	–

* Column percentages reported, that is, percentage of total responses.

† Row percentages reported, stratified by proxy response status.

‡ Individuals in Uganda were asked about albendazole, ivermectin, praziquantel, and zithromax separately. To avoid duplicate responses, only ivermectin responses were included.

### Unadjusted associations between proxy response status and covariates of interest.

To assess proxy response status directly, we first modeled unadjusted associations between proxy response status and eligibility to participate in the MDA, categories of age, sex, district of subject, and country of subject. The dataset used for this analysis included all adults (ages > 10) regardless of eligibility status, resulting in 6,261 subjects. The unadjusted odds of proxy response status were statistically significant for eligibility, categories of age and sex (Table 3). Unadjusted odds of proxy response status were also significant among Diebougou (Burkina Faso) and Machinga (Malawi) residents, as well as by country. The odds of requiring a proxy response was 3.55 (95% CI: 2.42, 5.21) times greater in eligible subjects compared with those ineligible. Adolescents had the greatest odds of requiring a proxy response, followed by young adults; older adults ages ≥ 40 were the least likely to require a proxy response. Compared with women, men had significantly higher odds of requiring a proxy response (1.89, 95% CI: 1.71, 2.10). The odds of proxy response varied significantly by country. Participants in the Malawian coverage surveys had the lowest odds of requiring a proxy response 0.59 (95% CI: 0.53, 0.66), while participants in the Burkinabe coverage surveys had the greatest odds of a proxy response 2.30 (95% CI: 2.07, 2.56), as compared with participants from Uganda.

**Table 3:** Unadjusted and adjusted odds ratios (OR), with 95% confidence intervals (CI), for proxy response status, by key covariates (eligibility, age, sex, and country) (*N* = 6,261)*

	Unadjusted		Adjusted†	
	OR	95% CI	OR	95% CI
Eligible				
Yes	3.55‡	(2.42, 5.21)	2.57‡	(1.55, 4.26)
No	Ref		Ref	
Age, years				
Older adults, age 40+ years	0.71‡	(0.63, 0.80)	0.43‡	(0.30, 0.61)
Young adults, age 19 to 39 years	0.79‡	(0.71, 0.88)	0.59	(0.35, 0.98)
Adolescents, age 11 to 18 years	Ref		Ref	
Sex				
Male	1.89‡	(1.71, 2.10)	1.98‡	(1.38, 2.85)
Female	Ref		Ref	
Country*				
Burkina Faso	2.30‡	(2.07, 2.56)	–	–
Malawi	0.59‡	(0.53, 0.66)	–	–
Uganda	Ref		–	–
District*				
Batie, Burkina Faso	1.05	(0.90, 1.23)	–	–
Diebougou, Burkina Faso	4.54‡	(3.88, 5.31)	–	–
Machinga, Malawi	0.73‡	(0.62, 0.86)	–	–
Zomba, Malawi	1.17	(1.00, 1.37)	–	–
Amuru, Uganda	Ref		–	–

* Country and district were included in the final model by including a random intercept term that adjusted for the hierarchy of clusters nested within districts.

† Adjusted model included eligibility, categories of age, and sex.

‡ Statistically significant at the α = 0.05 level.

### Adjusted, hierarchically nested model for proxy response status.

We fit a generalized linear mixed model with random intercepts for cluster and district in our adjusted model, which included eligibility, age, and sex as fixed effects. A mixed model was necessary to account for the likely clustering of responses according to geography. We fit a multivariable model describing proxy response status, adjusting for eligibility, age categories, sex, and interaction terms for eligibility by sex and eligibility by age category. These multivariable models also included random intercepts for district and cluster. We performed tests for issues of collinearity and assessed the interaction terms through backward elimination. All interaction terms were nonsignificant and were dropped from the model. To test the covariates and the significance of random effects we included the option COVTEST in these models, both of which were significant. Our final model included eligibility, age category, sex, and the random intercepts (Supplemental A). According to our final model, the odds of requiring a proxy response was 2.57 (95% CI: 1.55, 4.26) times greater among eligible subjects compared with those ineligible (Table 3). Older adults had significantly lower odds (0.43, 95% CI: 0.30, 0.61) of requiring a proxy response compared with adolescents. Compared with women, male subjects had 1.98 (95% CI: 1.38, 2.85) times greater odds of requiring proxy responses.

### Unadjusted associations between drug coverage status and covariates of interest.

The final analytic model to assess drug coverage included 6,604 subjects, after excluding the 329 subjects who were reported as ineligible for MDA or were missing eligibility status, as ineligibility was a perfect predictor of drug coverage. The unadjusted odds of drug coverage were statistically significant for proxy response status, sex, and country (Table 4). The odds of reporting ingestion of the drugs was 1.70 (95% CI: 1.17, 2.46) times greater in subjects with a proxy response compared with self-reporters. Men had a significantly lower odds of reporting ingestion of the drugs compared with women (0.71, 95% CI: 0.62, 0.82). Residents of Burkina Faso and Malawi had 15.05 (95% CI: 11.24, 20.16) and 1.80 (95% CI: 1.55, 2.11) times the odds of having ingested the drugs compared with residents of Uganda. Similarly, residents of the specific districts in Burkina Faso and Malawi all had significantly higher odds of drug coverage compared with those residing in Amuru, Uganda. In the unadjusted analysis, age was not significantly associated with increased odds of drug coverage.

**Table 4:** Unadjusted and adjusted odds ratios (OR), with 95% confidence intervals (CI), for reported drug coverage, by key covariates (proxy response, age, sex, and country) (*N* = 6064)*

	Unadjusted		Adjusted†	
	OR	95% CI	OR	95% CI
Proxy response status				
Yes	1.87‡	(1.61, 2.16)	1.70‡	(1.17, 2.46)
No	Ref		Ref	
Age, years				
Older adults, age 40+ years	1.12	(0.96, 1.31)	0.74	(0.42, 1.32)
Young adults, age 19 to 39 years	0.89	(0.78, 1.02)	0.82	(0.56, 1.19)
Adolescents, age 11 to 18 years	Ref		Ref	
Sex				
Male	0.71‡	(0.62, 0.82)	0.57‡	(0.33, 0.98)
Female	Ref		Ref	
Country*				
Burkina Faso	15.05‡	(11.24, 20.16)	–	–
Malawi	1.80‡	(1.55, 2.11)	–	–
Uganda	Ref		–	–
District*				
Batie, Burkina Faso	62.52‡	(35.12, 111.30)	–	–
Diebougou, Burkina Faso	21.20‡	(15.08, 29.82)	–	–
Machinga, Malawi	5.44‡	(4.41, 6.72)	–	–
Zomba, Malawi	5.12‡	(4.14, 6.633)	–	–
Amuru, Uganda	Ref		–	–

* Country and district were included in the final model by including a random intercept term that adjusted for the hierarchy of clusters nested within districts.

† Adjusted model included proxy response status, categories of age, and sex.

‡ Statistically significant at the α = 0.05 level.

### Adjusted, hierarchically nested model for drug coverage status.

To adjust for covariates of interest (proxy response, age, and sex) while taking into account the likely clustering of responses according to geography, we again fit a generalized linear mixed model with random intercepts for cluster and district. We proceeded with model selection and analyzed model fit in the same fashion as our models for proxy response status. We fit multivariable models with drug coverage as the dependent variable and independent variables for proxy response status, age category, and sex, as well as interaction terms for proxy response by sex and proxy response by age category, keeping the random intercepts for district and cluster. After testing for issues of collinearity, we assessed the interaction terms through backward elimination. All interaction terms were nonsignificant and were dropped from the model. We also tested the independence of the covariates and the significance of random effects using COVTEST, both of which were significant. Our final model included proxy response status, age category, sex, and the random intercepts (Supplemental B).

In our final model, subjects with a proxy response had a significantly increased odds of ingesting the drugs, compared with self-reporters (OR: 1.70, 95% CI: 1.17, 2.46), after controlling for age categories and sex. Male subjects had significantly lower odds (0.58, 95% CI: 0.36, 0.93) of ingesting the drugs compared with female subjects (Table 4). Age categories were not significantly associated with the odds of drug coverage in the adjusted model.

## DISCUSSION

Proxy responses can often pose problems in survey design. Choosing to include proxy responses is convenient—they reduce nonresponse rates and, in some cases, reduce the cost of data collection—but previous research has not produced conclusive evidence to suggest that proxy responses are accurate in their ability to approximate self-reporting.^13^ This study quantified the association between proxy response status and drug coverage among persons receiving preventative chemotherapy and is the first published study to directly assess proxy reporting during NTD coverage surveys. By including data from coverage surveys in three distinct countries, Burkina Faso, Malawi, and Uganda, this study was able to capture some of the programmatic and cultural diversity found across NTD programs, suggesting that the significant associations found in this study may be generalizable to other settings.

In particular, this study found that the odds of ingesting MDA drugs were 1.7 times greater in proxy respondents compared with self-reporters, after adjusting for age and sex and including random intercepts for PSU and district. To evaluate the characteristics of proxy responses, we modeled covariates of interest in unadjusted and adjusted hierarchical models. Age, sex, and eligibility for MDA were all strongly associated with proxy responses in unadjusted models. Requiring a proxy response was also strongly associated with district and country: the odds of subjects requiring a proxy response in Diebougou, Burkina Faso, was much higher compared with subjects, in Amuru, Uganda. To account for the likely correlation between subjects living in the same clusters within the same countries, a hierarchical, multivariable model was fit. In this model, eligible subjects and men had greater odds of requiring a proxy response compared with ineligible subjects and women. Taken together, these results suggest that differences in coverage survey implementation, including prior social mobilization to sensitize the population in advance of the coverage survey administration, the timing of the survey, and the persistence of the survey team to follow up when people are absent, can have a significant effect on the proportion of proxy responses.

The differences between men and women within specific age groups could be explained, in part, by educational attainment and employment practices. Men are more likely to work outside the home, and are more likely to pursue education for longer periods than women.^14^^–^^16^ Therefore it follows that men are less likely to be present for a population-based survey and are more likely to require a proxy response. By this same logic, it is not unreasonable to presume that individuals who were not available at the time of the coverage survey are also less likely to have been available for the actual MDA, suggesting that one might expect coverage rates to be lower in individuals with a proxy response compared with self-report. It is difficult to determine what drove the differences in proxy response by district and country. Possible factors include the timing of the survey (e.g., if it occurred during a period of seasonal work outside the home), the effectiveness of community mobilization leading up to the survey, and the persistence of the field teams to revisit households where individuals were missing.

Our work suggests that proxy respondents may be more likely to report that the subject for whom they are responding ingested the drug(s) compared with individuals responding for themselves. Recent work exploring childhood vaccination status and maternal report have suggested high concordance between maternal recall and vaccination records, but also indicate that maternal recall may overestimate true vaccination coverage.^17^^,^^18^ Our work echoes these findings, but indicate a more substantial overestimation of coverage than previously reported. This could be in part due to social desirability bias among proxy respondents, who in this case, know that swallowing the drugs is the desired behavior and therefore report ingesting the drugs more frequently.^13^ In the present study, we assessed drug coverage in primarily low-income settings, which may also have impacted our results. A study assessing NTD control measures in other countries suggests that the effect of social desirability bias within vulnerable communities is particularly pronounced.^19^ Within this research setting, study respondents may have felt that responding negatively with regards to the MDA program, indicating that drugs were not received or not ingested, could impact their future treatment or the employment of the community drug distributer. The authors did not have access to a written register to compare proxy responses to self-reported responses and, therefore, we were unable to assess the validity or concordance of responses. For this reason, we cannot rule out the possibility that the proxy responses accurately represent MDA compliance for the absent individuals. While there are child vaccination studies to indicate that concordance between maternal proxy responses and a written record is high, there is also evidence of an overestimation of vaccination status among maternal recall, compared with written records.^17^^,^^18^

A major limitation of this study is the lack of a register of drug coverage to validate proxy responses or assess concordance. Further studies that directly measure the concordance between self-reported drug coverage and proxy-reported coverage, using written registers, would be important to determine the reliability of proxy-reported responses. An additional challenge was the fact that the coverage questionnaires differed across the three countries and not all datasets retained a household level indicator, making it impossible to account for household-level correlation in the models.

Despite its limitations, this study provides several important insights. First and foremost, age, sex, and eligibility to receive preventative chemotherapy medications are strongly associated with whether an individual is unavailable at the time of a survey and requires a proxy response. Future studies may also consider contacting respondents that are absent at the time of an MDA program or drug coverage survey through alternate means (e.g., via cellphone or email) before relying on a proxy response, given the ubiquity of this technology in study areas. Though there is little published data to support the use of cellphones as a tool to decrease proxy responses, the authors are aware of cellphones being used successfully in the context of the Supervisor’s Coverage Tool to obtain firsthand responses from participants who are not at home.

These findings may also have important implications to other population-based surveys, particularly the impact assessments that are used to make stop MDA and surveillance decisions. If individuals are unavailable during MDA are also unavailable at the time of the assessment, the resulting program conclusions may be overly optimistic. In other words, this study calls into question whether statistically rigorous population-based surveys, which are a staple of NTD programs, produce unbiased results. Further research to understand whether NTD surveys are inadvertently missing populations who are at greater risk of disease because of systematically missing treatment is urgently needed. Alternative tools, including qualitative research techniques and respondent-driven sampling, may provide useful insight into which populations are being missed and how to better reach them.

Ultimately, the WHO’s goals for NTD control and elimination are contingent upon the MDA programs achieving adequate drug coverage. Population-based coverage surveys are a good tool for validating administrative coverage estimates and independently assessing if effective coverage has been achieved. These surveys often rely on proxy responses to save time and avoid the bias that would be induced if these individuals were skipped altogether; however, accepting proxy responses is only an effective strategy if the responses are a valid surrogate for what an individual would otherwise self-report. It is important to understand how proxy responses differ from self-report because proxy responses can comprise a large proportion of the overall survey responses—in this study, 39.2% of responses for persons > 10 years were provided via proxy—and therefore any systematic differences may induce bias in the overall coverage estimate. With NTD programs driving toward elimination, more and more countries are expected to implement coverage surveys to verify the effectiveness of the MDA. Determining the reliability of proxy responses will be crucial for identifying best practices around NTD coverage surveys. In the meantime, we recommend that survey implementers take steps to minimize the need for proxy responses by conducting coverage surveys at a time of the year (and day) when most people are home, working with local leaders to ensure the communities are aware of and sensitized to the survey in advance, and using cellphones to garner firsthand responses from absent individuals.

## Supplemental tables


Supplemental materials
