# Supplementary material for: Proxy Responses for Mass Drug Administration Coverage Surveys: The Trends and Biases When Others are Allowed to Respond
Source: Am J Trop Med Hyg. 2021 Oct 25;106(1):268–74. doi: 10.4269/ajtmh.21-0817 (PMC8733507; doi:10.4269/ajtmh.21-0817)
Supplement: Supplementary file 1 [file tpmd210817.SD1.pdf]

Supplemental File

Model A: Final model for proxy response status:

$$\text{Logit}(E(Y_{ijk}|b_{jk}) = \beta_0 + \beta_1(\text{Eligibility Status})_{ijk}$$

$$+ \beta_2(\text{Older Age Category})_{ijk}$$

$$+ \beta_3(\text{Young Adult Age Category})_{ijk}$$

$$+ \beta_4(\text{Sex})_{ijk}$$

$$+ b_0(\text{PSU})_{jk}$$

$$+ b_1(\text{District})_k$$

$$+ e_{ijk}$$

For *i* individuals in *j* primary sampling units of *k* districts, where Yijk = 1 if the individual had a proxy response and 0 for self-response

Model B: Final model for drug coverage:

$$\text{Logit}(E(Y_{ijk}|b_{jk}) = \beta_0 + \beta_1(\text{Proxy Response Status})_{ijk}$$

$$+ \beta_2(\text{Older Age Category})_{ijk}$$

$$+ \beta_3(\text{Young Adult Age Category})_{ijk}$$

$$+ \beta_4(\text{Sex})_{ijk}$$

$$+ b_0(\text{PSU})_{jk}$$

$$+ b_1(\text{District})_k$$

$$+ e_{ijk}$$

For *i* individuals in *j* primary sampling units of *k* districts, where Yijk = 1 if the individual is reported to have swallowed the drug, 0 if not.
